# ODEP-Based Robotic System for Micromanipulation and In-Flow Analysis of Primary Cells

**DOI:** 10.34133/cbsystems.0234

**Published:** 2025-03-06

**Authors:** Joanna Filippi, Paola Casti, Valentina Lacconi, Gianni Antonelli, Michele D’Orazio, Giorgia Curci, Carlo Ticconi, Rocco Rago, Massimiliano De Luca, Alessandro Pecora, Arianna Mencattini, Steven L. Neale, Luisa Campagnolo, Eugenio Martinelli

**Affiliations:** ^1^ Department of Electronic Engineering, University of Rome Tor Vergata, 00133 Rome, Italy.; ^2^ Interdisciplinary Center for Advanced Studies on Lab-on-Chip and Organ-on-Chip Applications (ICLOC), 00133 Rome, Italy.; ^3^ Department of Biomedicine and Prevention, Tor Vergata University, 00133 Rome, Italy.; ^4^ Department of Surgical Sciences, Section of Gynecology and Obstetrics, University of Rome Tor Vergata, Rome, Italy.; ^5^ Department of Gender, Parenting, Child and Adolescent Medicine, Physiopathology of Reproduction and Andrology Unit, Sandro Pertini Hospital, Rome, Italy.; ^6^ Italian Nation Research Council (CNR), Rome, Italy.; ^7^ James Watt School of Engineering, University of Glasgow, Glasgow, UK.

## Abstract

The presence of cellular defects of multifactorial nature can be hard to characterize accurately and early due to the complex interplay of genetic, environmental, and lifestyle factors. With this study, by bridging optically-induced dielectrophoresis (ODEP), microfluidics, live-cell imaging, and machine learning, we provide the ground for devising a robotic micromanipulation and analysis system for single-cell phenotyping. Cells under the influence of nonuniform electric fields generated via ODEP can be recorded and measured. The induced responses obtained under time-variant ODEP stimulation reflect the cells’ chemical, morphological, and structural characteristics in an automated, flexible, and label-free manner. By complementing the electrokinetic fingerprint of the cell centroid motion with data on the dynamics of electro-deformation and orientation, we show that subtle differences at the single-cell level can be elucidated. Specifically, here, we demonstrate, for the first time, the ability of the combined ODEP-based robotic and automatic analysis platform to discriminate between primary endometrial stromal cells obtained from fertile patients and patients with disrupted receptivity/selectivity equilibrium. When multiple cells were considered at the patient level, the performance achieved an average accuracy of 98%. Single-cell micro-operation and analysis systems may find a more general application in the clinical diagnosis and management of patients with pathological alterations at the cellular level.

## Introduction

The characterization of cell phenotypes is crucial in medical tests and in drug efficacy evaluation, especially in the presence of multifactorial diseases or enigmatic disorders [1]. In such scenarios, the absence of specific gene patterns or individual triggering factors linked to the pathology requires a laborious, costly, and time-consuming combination of investigations and searches for plausible clues, defining cell signatures or fingerprints, i.e., comprehensive yet distinguished representations of abnormal cell profiles [2]. Biophysical approaches to the analysis of cells are gaining much attention in light of the possibility of investigating cell processes with high precision at the micro- and nanoscale levels and in stimulus–response scenarios, including electromagnetic, chemical, or mechanical influences. Techniques such as micropipette aspiration [3], atomic force microscopy [4], Raman spectroscopy [5], or tweezers exploiting optical [6], optoelectronic [7,8], magnetic [9], and acoustic principles [10] offer unique insights and are continually evolving to provide data not only on the cell organization structure but also on the way cells sense and respond to the surrounding microenvironment.

Paired with the analytical capabilities of the methods, devising an automated micro-operation system is also pivotal to facilitate efficient management of the pipeline for cell analysis [11]. Automation of operations for cell manipulation, stimuli devising, response monitoring, and characterization improves the throughput and usability of the system, reducing the confounding effects of manual and user-dependent contributions. Existing methods to tackle these objectives possess limitations. Most of them have limited temporal or spatial resolution or do not possess the reconfiguration properties required to be extended to multiple biological experimental domains [10]. For example, atomic force microscopy is a destructive method requiring time-consuming operations. The advantage, in this case, is the derivation of an accurate estimate of the cell’s local properties in relation to the deflection of the cantilever. However, destructivity and low throughput, which also hold for other standard techniques like micropipette aspiration, do not allow the quantification of multiple cell properties at the same time or at diversified stimuli conditions, neither the measurement of multiple cells in parallel. These characteristics might limit the discriminative potential of those techniques in the presence of multifactorial defects, where the nature of the differences among conditions might not be univocal or a priori known. Existing microfluidic-based approaches like optical, optoelectronic, and acoustic tweezers, and mechanical methods can provide the required throughput and experimental environment for multivariate analysis. Differences hold regarding the spatial resolution, which are in the range of (1 to 10 μm) for acoustic, hydrodynamic, and optoelectronic tweezers, while reaching (0.1 to 1 nm) for the optical counterpart. The optical tweezer, although giving a higher spatial resolution, requires labeling for smaller particles, a high-numerical-aperture lens, and a high-powered laser (>10^5^ W/cm^2^), which can damage the cells [12,13]. Acoustic tweezer is a label-free method, functioning with a low-light power intensity (10^−2^ to 10 W/cm^2^), capable of sorting, patterning, and enriching bioparticles without the need for a low-conductivity media. However, this technology also brings some difficulties related to the choice of the suitable configuration for a specific application [12]. Mechanical methods based on microvalves, pumps, and microstructures provide cell parallel manipulation, sorting, and isolation based on size, shape, and deformability [14]. Differently, optoelectronic tweezers, also known as optically-induced dielectrophoresis (ODEP), exploiting a locally nonuniform electric field concentrated by virtual electrodes, provide powerful versatility and large forces with low light power intensity (10^−2^ to 10 W/cm^2^) [12]. This unique feature, paired with electrode reconfigurability, allows easy adaptation to diversified biological case studies and the elucidation of diversified local cell properties. Provided the extreme flexibility of projected light patterns, ODEP represents a powerful technology for single-cell manipulation and characterization. Studies in the literature have demonstrated incredible ODEP performance for the characterization of bacteria subclones with antibiotic resistance [15], manipulation and sorting of circulating tumor cells [16–18], recognition of cell types with different levels of drug resistance and viability [19], apoptosis levels [20] even at the very early stage [21], differentiation of cell populations and chemotherapy effects [22], as well as different transcript levels of selected genes [21]. These achievements have been sustained by technological developments in the microfluidics and microfabrication [23], allowing for handling cells in scenarios of lab-on-chips (LOCs). These devices provide an environment with controllable experimental conditions, e.g., laminar flow, possibility of serialization and parallelization of operations, integrability with live-cell microscopy, and machine learning modules [24–27].

Recently, ODEP has also allowed to extract a portrait of the cell heterogeneity in the presence of nonuniform electric fields [21,22]. In particular, with an in-flow ODEP platform [21], we demonstrated the close link between cell frequency-dependent dielectric characteristics and ODEP-induced motions, paired with the possibility to analyze single cells based on the electrokinetics of their centroid. When subject to ODEP between 2 alternatively active virtual electrodes at multiple frequency values, cells underwent oscillatory motions whose nonstationarity characteristics provided meaningful insights about their biological status. Cells, however, are neither rigid nor homogeneous bodies and respond depending on the local dielectric characteristics as well as on the deformability properties of the cell compartments [28,29]. Such electrodeformations arise theoretically from the Maxwell stress tensor, whose integral over the cell volume provides the resulting DEP force exerted on the cell centroid [28,30]. Viscoelastic properties of the cell membrane, resistance to stress due to the cytoskeleton structure, nuclear-cytoplasmic ratio, and organelle spatial arrangements all might contribute to define the characteristics of the local displacements over time [29]. Kinetics of cell deformations and orientations, along with the electrokinetic responses of the cell centroid, might open a new door for the study of cellular modifications to date not yet addressed.

With this in mind, in this work, we propose a robotic system for the in-flow micromanipulation, measurement, and analysis of single cells. The automated control system allows to fully harness the ODEP concept by projecting highly reconfigurable electrodes and by varying the stimuli characteristics over time. Such a system enables the collection and elucidation of a set of cell responses to better represent their phenotypes and so increase the analytical perspective. As stated above, being nonspherical and nonhomogeneous bodies, during the time course of the experiment, cells experience composite motions including translation, reorientation, and deformation, depending on the dielectric, viscoelastic, structural, and morphological properties of the cell compartment at the micro- and nanoscales. Therefore, in addition to the analysis of the cell centroid motion based on the wavelet-scattering transform (WST) [31,32], we extracted information on local cell electrodeformation via particle image velocimetry (PIV) [33] in a reference system integral with the cell centroid motion. The information derived from the vector fields on local magnitude and orientation responses over time was characterized. When subject to varying ODEP frequencies in an alternate electrode configuration, we show that indicators of regularity and of the characteristics of distributions of the time-varying deformation and orientation profiles provide valuable assessments to complement the cell phenotype. Unlike other methods existing in the literature [34], the proposed descriptors of the cell electrokinetic and electrodeformation responses are not conventional mechanical or dielectric parameters. This choice, while potentially a limitation, is also the key feature of the proposed approach and related to its peculiarities. In fact, the ODEP experimental setup is designed to facilitate parallelization and automation of the operations, with the possibility of performing in-flow measurements with minimal detrimental effects for the cells. Over the course of the experiment, cells undergo through ODEP stimuli of nonstationary nature and respond through a series of composite oscillatory motions that can be regarded as the combination of the centroid displacements accompanied by local deformations and reorientations. The time-varying stimuli depend not only on the applied frequency patterns but also on the gradient of the electric field experienced by the cell over the course of the experiment. Such gradient is spatially distributed nonuniformly in the chip, with magnitude and directions influencing the cell motion and depending, in turn, from the cell-specific positions occupied by the cells over time [21]. Additionally, as the cells are in suspension in a 3-dimensional (3D) chamber, variations in altitude (over the *z* direction) affect the displacement estimates on the projected 2D plane. Therefore, the electrokinetic and electrodeformation responses are not expected to be indicative in terms of inherent values, but rather in terms of dynamics of variations.

To fully assess the potential diagnostic and prognostic applications of the proposed approach, we investigated, for the first time, the electrokinetic patterns of patient-derived primary endometrial stromal cells in response to ODEP at the single-cell level, aiming to stratify patients according to their reproductive history. To this end, we enrolled in the study fertile patients and patients with 2 types of recurrent reproductive failure (RRF), namely, recurrent implantation failure (RIF) and unexplained recurrent pregnancy loss (uRPL) [35,36]. RIF and uRPL represent 2 conditions related to endometrial dysfunctions with opposite endometrial response [37]. Indeed, it has been widely demonstrated that RIF is associated with excessive endometrial selectivity, which prevents embryo implantation regardless of its quality; on the contrary, RPL occurs due to excessive endometrial permissiveness, allowing the implantation of embryos with poor developmental potential [38–40]. In 50% of RPL cases, the etiology of the miscarriage cannot be identified, classifying it as unexplained (uRPL).

Recognition of the pivotal role of the endometrium and endometrial receptivity in the etiopathology of both RIF and uRPL is gaining momentum, driving the search for early markers of these conditions. Numerous studies have sought to identify the immunological and molecular signatures of RIF and RPL [37,41–43]. Indeed, there is a clear consensus emerging that they represent distinct pathological conditions affecting the preparation of the endometrium to implantation (namely, decidualization) and both of which are the main causes of reproductive failure [39]. However, tools for the early diagnosis of these conditions remain scant and mostly reside on time-consuming and costly analyses. Omics studies have identified alterations of biological pathways and processes behind cell reprogramming during the process of decidualization, regulating cell motility, cell adhesion, cytoskeleton organization, extracellular matrix (ECM)–receptor interaction, and signal transduction in both RIF and RPL patients [36,38].

The primary cells analyzed are directly isolated from living tissue. These cells, unlike immortalized cell lines, retain the characteristics of their donors, with differences in gene expression, protein function, and cellular behaviors, including metabolic states and responses to the microenvironment. As a consequence, the cells possess an inherent heterogeneity in terms of dimensions and morphology variability (reflected at the level of electrokinetic responses) that not only vary over the course of the time lapse in response to the electric field but also are intra-patient and transversal to the categories analyzed. This motivates the need for a broader and multivariate analytical perspective based on indicators of nonstationarity, regularity, and statistical nature.

We demonstrate that endometrial cells derived from fertile, RIF, and uRPL patients exhibit different dielectric responses, not only in terms of electrokinetics of the cell centroid but also in terms of cell electrokinetics of deformation and orientation. These pieces of information may complement standard biological investigations to allow discrimination of the 3 conditions. In this respect, we confirmed the ODEP results by gene and protein expression analysis. Our results demonstrate that the proposed system, which facilitates multiple measurements through automated operations, provides quantitative signatures of ODEP-induced cell responses and, through machine learning of cell electrokinetic profiles, could facilitate patient stratification in clinical practice.

## Materials and Methods

### Experimental design

**Fig. 1.**
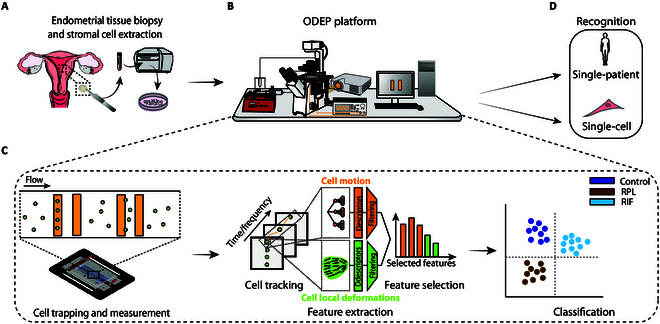
Workflow of the proposed approach. (A) Endometrial biopsy taken from patients. Endometrial stromal cells were isolated and cultured. (B) ODEP platform for single-cell manipulation and measurement with the control system and the instrumentation. (C) Trapping, measurement, and analysis steps. (D) Recognition of single cell and single patient.

In this study, endometrial stromal cells were extracted to investigate clues of the presence of pathologies (RIF and uRPL) related to the level of selectivity or receptivity of the endometrium. Primary cells are obtained from a patient’s tissue biopsy (Fig. 1A). After cell expansion, twin dishes are devised to be analyzed in parallel with the proposed system and with reverse transcription polymerase chain reaction (RT-PCR) for validation purposes. The ODEP platform allowed the cell trapping and measurement through an automatic control system interface, including a graphical user interface (GUI) and instruments such as an inverted microscope, a function generator, a projector, and a syringe pump (Fig. 1B). Cells were suspended in a low-conductivity buffer, loaded into the LOC device, trapped, and measured under continuous flow by means of rectangular projected electrodes. As shown in Fig. 1C, the cells bounce between 2 virtual electrode barriers, operating in an alternate mode by varying the frequency values of the input voltage. Videos of cell motions are recorded via time-lapse microscopy (TLM) not only during the application of ODEP stimuli but also during the transitory periods between one frequency and the next. The centroid of single cells is tracked over time, and the cell motion area is automatically segmented.

The multi-frequency composite motions experienced by the cells are characterized, globally, relative to the cell centroid and, locally, with the extraction of vector fields of local displacements over time in the reference system integral with the centroid. Descriptors of trajectories are obtained via WST (electrokinetics of the cell motion), while statistical and regularity descriptors are extracted from local displacement signatures (kinetics of cell deformations and orientations at different stimulation frequencies). Relevant descriptors are automatically selected with a filtering step followed by sequential feature selection (SFS) [44], and used in a supervised classification model to discriminate between physiological and pathophysiological cell and patient states (Fig. 1D).

### Experimental setup

**Fig. 2.**
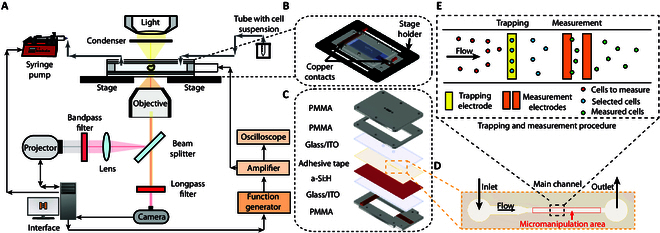
Overview of the ODEP-based manipulation and measurement platform. (A) Manipulation, optical, and microfluidic system setup. (B) Detail of the chip located on the microscope stage. (C) Exploded view of the chip with details of the different layers and constituting materials. (D) Detail of the chip’s channel constituted by an inlet, an outlet, and a trapping/measurement area. (E) Trapping and measurement procedure of the single cells.

The setup is constituted by manipulation, optical, and microfluidic systems, and it is shown in Fig. 2A. An ODEP-based chip (Fig. 2B) was located on the stage of an inverted microscope (Leica DMi8, Leica Microsystems CMS GmbH, Wetzlar, Germany) and connected to an amplifier (WA301 Wide Band Amplifier, TTi, UK) and a function generator (33120A Hewlett Packard, USA). The light pattern was generated by a digital light processing (DLP) projector (EH334 Optoma, Taiwan), filtered by a band-pass filter (FB600-40, Thorlabs Inc., USA), focused by a biconvex lens (LB1945-A, Thorlabs, USA), and conveyed through a 20× objective toward the chip via a beam splitter. The band-pass filter allows the transmission to the chip of only the wavelengths that photoactivate the amorphous silicon (a-Si), while the others are cut off. This configuration avoids the increase of the power transmitted to the cell medium and limits the warming and evaporation and, as a consequence, possible stress or damage to cellular structures [22]. A long-pass filter (FEL0650, Thorlabs Inc., USA) located in front of the camera prevents the saturation. A syringe pump, operating in withdraw mode, draws the cell suspension from a tube toward the ODEP chip at 0.5 μl/min. The cells were suspended in sucrose-based buffer, i.e., double-distilled water, 8.5 wt % sucrose, 0.5 wt % bovine serum albumin (BSA), and 1% (v/v) phosphate-buffered saline (PBS 1×) with a conductivity of 15 mS/m. For further details, please refer to our previous works [21,22].

### Design and fabrication of the ODEP-based device

The ODEP-based device was designed with Autodesk Inventor and cut by means of a laser cutter (Speedy 100, Trotec Laser Inc., Austria) [21]. It includes different layers as shown in the exploded view of Fig. 2C. The heart of the device is the chamber constituted by a channel (*L*: 12 mm, *H*: 60 μm) inserted between 2 layers of indium tin oxide (ITO)–coated glass (*L*: 75 mm, *W*: 25 mm, *H*: 1 mm) (15 to 25 ohms/sq, Sigma-Aldrich, St. Louis, MO, USA), one of them coated by 600-nm-thick a-Si through plasma-enhanced chemical vapor deposition (PECVD). The width of the micromanipulation area is equal to 0.8 mm (Fig. 2D). The chamber is located on the polymethyl-methacrylate (PMMA) base (TroGlass Clear, Trotec Laser Inc., Austria) in turn fasted to microscope stage insert (PeCon GmbH, Germany). Finally, the top part includes the fluidic connections realized by means of 2 PMMA layers. For further details on the fabrication process, please refer to Section S1.

### Robotic system for cell micromanipulation and measurement

**Fig. 3:**
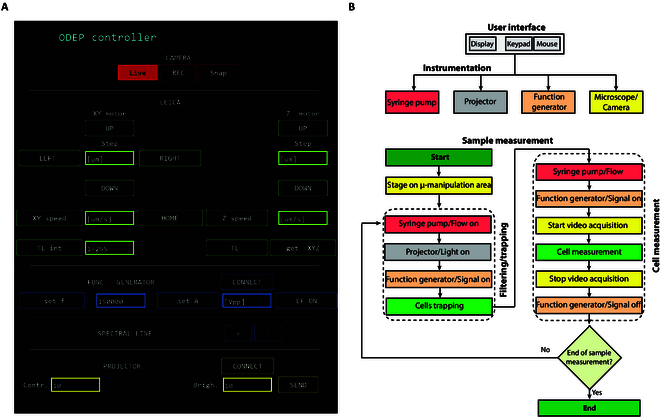
The robotic system. (A) Image of the system interface. (B) Flow chart with the sequence of the sample measurement steps provided by the software.

The cell micromanipulation and measurement are schematized in Fig. 2E and were performed by means of our custom control system using an interface realized in Python (v.3.6.5) [21,22] properly modified for this application. As shown in Fig. 3A, the robotic system is controlled via a simple and interactive GUI constituted by the instruments panel, the live window showing the current field of view with the superimposed virtual pattern, and the projection window with the light pattern, allowing to easily adapt the experimental procedure according to specific needs. The flow chart in Fig. 3 shows the entire control system with the user interface, the instrumentation (Fig. 3B), and the experimental procedure (Fig. 3C).

At the beginning of the session, the stage of the microscope is located in correspondence of the micromanipulation area of the ODEP-based device. The cell suspension is loaded into the channel at 0.5 μl/min. Two rectangular electrodes (*L*: 40 μm, *W*: 800 μm), at a distance of 60 μm each other, are projected to work as a filter to entrap multiple cells under continuous flow at 10 V_pp_ and 150 kHz.

After the trapping, the flow is stopped and the cells are measured with a pair of virtual rectangular electrodes (*L*: 40 μm, *H*: 300 μm) located at a distance of 60 μm from each other center. A signal of 10 V_pp_ is applied to each electrode in an alternate way at 26 decreasing frequency values from 150 to 25 kHz with a step of 5 kHz, which avoid side effects related to electrolysis. In detail, the cells are aligned between the 2 electrodes, and, at the first step, the video recording starts with the acquisition of the cell motion induced by the activation of the first virtual electrode. After 1s, the signal is turned off and the video recording is stopped.

In the second step, the video recording starts, and the other electrode is activated at the subsequent frequency. After 1 s, the video acquisition is stopped, and the electrode is turned off. The procedure is repeated automatically for all 26 frequencies by maintaining the fixed amplitude at 10 V_pp_. The procedure lasts for around 50 s, thus reducing the possibility that changes in the physical properties of cells happen during measurements.

**Fig. 4.**
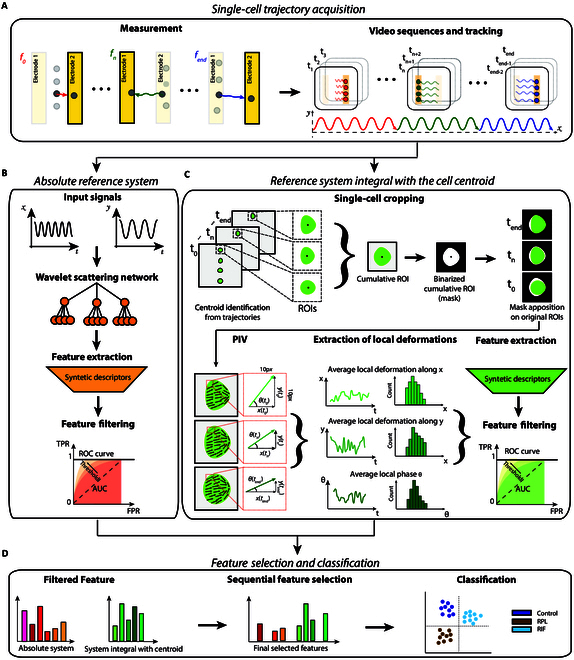
Schematic representation of the proposed method. (A) Single-cell trajectory acquisition with the measurement of the cells at the diverse frequencies, the video recording, and the cell tracking. (B) Derivation of motion descriptors from cell centroid trajectory in absolute reference system. (C) Derivation of descriptors of electrokinetics of deformation and orientation in the reference system integral with the cell centroid. (D) Feature filtering and selection from the 2 reference systems and classification results.

Videos at each frequency are acquired at 20 fps and at a spatial resolution of 0.45 μm/px. A simplified scheme of the measurement procedure is shown in Fig. 4A. The switch between the 2 mirrored electrodes induced an oscillating motion under positive dielectrophoretic force (pDEP), a steady state in correspondence of the crossover frequency, and a straight motion under negative dielectrophoretic force (nDEP). This behavior depends on the applied signal and on the dielectric and morphological properties of the cells, as also demonstrated in our previous work [21]. The ODEP-based trapping is shown with a video example in Movie S1.

### Derivation of motion descriptors from electrokinetic responses of the cell centroid (absolute reference system)

In the absolute reference system, WST-based descriptors of the electrokinetic responses are extracted from the trajectories of the cell centroid motion [21].

#### Extraction of cell centroid trajectories

For each cell, the obtained 26 video clips, one for each ODEP frequency value, are concatenated to obtain a unique video including all the applied frequencies. The cell trajectories are automatically extracted by means of the proprietary software Cell Hunter previously validated in our works [45–48] as shown in Fig. 4A. For the subsequent analysis, videos of duration *T* = 50 s (1,000 frames) were considered as representative of the centroid motion of each cell. In this manner, we mainly considered the oscillating motion confined between the 2 electrodes in the range [55 to 150] kHz. More details are included in Section 2.

#### WST-based descriptors of centroid motion

Following the same procedure of our previous work [21], a set of 568 descriptors is extracted from the cell centroid trajectories, as illustrated in Fig. 4B and summarized in Table S1. As already shown for other applicative scenarios [21], relative displacements of the cell centroid along the *x* and *y* directions might represent an important source of information related to the dielectric and morphological characteristics of the cell, depending on the applied frequencies. This information is extrapolated by means of the WST [31,32] through a cascade of signal decomposition that characterizes the nonstationarity of the cell motion [21]. The central frequency values of the 2-order filter banks used for the WST cover the range [0.06 to 9.1] Hz and [0.07 to 8.3] Hz, respectively. For further details, please see Section S3.

### Derivation of local motion descriptors from electrokinetic responses of deformation and orientation (relative reference system)

In the reference system integral with the centroid motion (relative reference system), local cell displacements are extracted via PIV to derive information on the electrokinetics of deformation and orientation over the time course of the experiment.

#### Segmentation of the cell motion area

The area containing all possible cell deformations over time is extracted to limit the analysis on the cell of interest and avoid considering displacements of neighboring cells or confounding elements. This step is also pivotal to ensure that the evaluation of the 2D deformation field is performed on the same area over time but in a reference system integral with the cell centroid. As the cells are in suspension in a 3D chamber, variations in altitude (over the *z* direction) correspond to variations of the refraction patterns and, consequently, of the imaged cell intensity values. In and out of focus areas, depending on the displacements along *z*, i.e., depth of correlation effect [49], and related to cell inhomogeneity, contributed differently to the displacement estimates, providing additional complementary information on the cell status. One of the effects of the cell motion in altitude is the large variations of the low-intensity profiles at the periphery of the cell, in correspondence with the cell membrane, which becomes more or less distinct or extended in relation to the cytoplasmic area, also in relation to the out-of-focus effects. Given the nontrivial segmentation scenario investigated, several existing segmentation methods were tested and failed in their purpose. Therefore, we decided to overcome the need of performing preliminary steps of image restoration, for counteracting the presence of blurring, or training steps with corresponding required labeling and validation procedures. We changed the point of view and addressed the detection of a motion area surrounding the cell, corresponding to the neighboring region in which deformation happen over time and that, cumulatively, include constrains on the presence of cell compartments (cytoplasm and boundary) relative to a single cell over time. This approach does not require segmentation of the cell boundary and resulted in a less accurate but robust delineation of the area of interest. The segmentation of the motion area, in fact, represents a step aimed at filtering the displacement fields extracted via PIV with a fixed area over time for subsequent analysis, and it is not used to characterize the properties of the cell boundaries or to track the cell motion itself. Therefore, also given the insufficient amount of available data to train deep learning architectures for the scope, an ad hoc adaptive algorithm was designed to segment the cell motion area needed for the analysis of the electrodeformation responses.

The proposed approach checks for the peripheral low-intensity boundary region and the cytoplasmic area and varies a segmentation threshold until their simultaneous presence is guaranteed. This encourages a more robust segmentation of the overall cell motion area. The steps of the abovementioned algorithm are schematized in Fig. 4C and described below.

##### Step 1: Derivation of a cumulative region of interest

Given the cell centroid coordinates, ***c*** $\left(t_{i}\right)$ = ($x\left(t_{i}\right),y\left(t_{i}\right)$), at time $t_{i}$ a squared region of interest (ROI), *ROI_ti_(*n,m*)*, [151 × 151] pixels ([68 × 68] μm) centered at ***c*** is extracted for each cell. Examples of regions of interest containing a single cell relative to different patients from each of the 3 biological categories (A) CTRL, (B) RIF, and (C) uRPL are shown in Fig. S1. In order to derive a 2D motion area including all possible displacements of the cell over time, a cumulative response is obtained, after histogram stretching of individual ROIs, by summing up, pixel by pixel, the regions of interest obtained for each cell over time, $t_{i}$, with *i* = 1, 2, …, *T* as:

$$\;I_{\mathrm{cum}}\left(n,m\right)=\sum_{i=1}^{T}\frac{{\mathrm{ROI}}_{t_{i}}\left(n,m\right)-\eta_{1}}{\eta_{99}-\eta_{1}}$$ (1)

with $\eta_{1}$ and $\eta_{99}$ indicating the 1st and 99th percentiles, respectively. The maximum and minimum values of pixels in the obtained image are finally scaled in [0 1].

##### Step 2: Initialization of a circular binary mask

A base binary image, ${\mathrm{bw}}_{\text{circ}}\left(n,m\right)$*,* is defined, for each cell, as a circular object centered at ***c*** with radius equal to the radius estimated by the circular Hough transform (CHT) at the first frame of the acquisitions. A preliminary binarization of $I_{\mathrm{cum}}$, ${\mathrm{bw}}_{th}\left(n,m\right)$*,* is performed with a threshold $\mathrm{th}={\mathrm{th}}_{O}$ set with the Otsu’s method [50] and used, in combination with the ${\mathrm{bw}}_{\text{circ}}\left(n,m\right)$, to derive initial estimates of 3 reference masks, as follows:

$${\mathrm{bw}}_{\text{cell}}\left(n,m\right)=\left({\mathrm{bw}}_{th}\left(n,m\right)\;\cap_{{\mathrm{obj}}_{m}}\;c\right)$$ (2)

$${\mathrm{bw}}_{\mathrm{cyt}}\left(n,m\right)=\left(\bar{{\mathrm{bw}}_{th}}\left(n,m\right)\;\cap_{{\mathrm{obj}}_{m}}\;c\right)\cap {\mathrm{bw}}_{\text{circ}}\left(n,m\right)$$ (3)

$$\;{\mathrm{bw}}_{\text{bound}}\left(n,m\right)={\mathrm{bw}}_{\text{cell}}\left(n,m\right)\cap {\bar{\mathrm{bw}}}_{\mathrm{cyt}}\left(n,m\right)$$ (4)

with the symbol $\bar{.}$ indicating the complement of the binary image and $\cap_{{\mathrm{obj}}_{m}}\;c$ the intersection operator restricted to the object with the maximum area containing c. At each step, the hole-filling operator is applied to all binary images and only the regions with the highest area not connected to the border of the frame were considered.

##### Step 3: Iterative refinement of the segmentation

Given the masks initialized at step 2, the effective segmentation of the boundary region is verified with the following conditions: $\mathrm{sum}\left({\mathrm{bw}}_{\text{bound}}\right)\neq 0$ and ${\mathrm{bw}}_{\text{bound}}\neq {\mathrm{bw}}_{\text{cell}}$. The condition is verified for most of the cell; if not, step 2 is repeated until the conditions are fulfilled with decreasing values of the threshold $\mathrm{th}=p\cdot {\mathrm{th}}_{O}$, with p=1−n∆p, ∆p=0.1, n=1,2,…,10 and then increasing values of the threshold with p=1+n∆, with n ϵ ℕ and th <1. The procedure is arrested in case the effective segmentation of the boundary region with all checked variations is not achieved. In this case, the cell motion area, ${\mathrm{bw}}_{\text{cell}}\left(x,y\right)$, is computed with Eq. (13) and $\mathrm{th}={\mathrm{th}}_{O}$*.*

#### Extraction of electrokinetics of cell deformations and orientations

In order to estimate cell deformations and orientations over the time course of the experiment, an optical method based on the PIV was chosen in light of the possibility of deriving vector fields that vary in space and time at appropriate resolutions [49]. PIV is conventionally used in a wide variety of aero and fluid dynamic applications, allowing the estimation of velocity of particles suspended in fluids. In light of the similarities in appearance, PIV is extended, in this work, to the study of cell local deformations in a reference system integral with the cell centroid. In PIV analysis, plausible local displacements between pairs of frames are evaluated based on the peaks of 2D cross-correlations. To the scope, uniform grids composed of interrogation windows are used to derive a vector field of displacements at time $t_{i}$, with $\begin{array}{c} i=1,2,\ldots ,T,U_{t_{i}}=(u_{t_{i}}\left(n,m\right),v_{t_{i}}\left(n,m\right)) \end{array}$, with *n* = 1, 2, …, *N*, *m* = 1, 2, …, *M* (*N*, *M* depending on the grid resolution).

To perform PIV analysis, some of the PIVlab algorithms [51,52] were selected and integrated in an ad hoc analysis workflow to balance the accuracy of the estimated displacements and the processing time. Individual ROIs are preprocessed with linear intensity mapping followed by contrast limited adaptive histogram equalization (CLAHE). Local displacement fields are evaluated between pairs of consecutive ROIs of the same cell for all frames, with a window size of 10 pixels (4.5 μm) corresponding to around ^1^/_10_ of the average cell diameter. Displacements at subpixel precisions are derived with the Gaussian 2 × 3 point fitting (3 points per each dimension) and 2 passes of fast Fourier transform (FFT) with bilinear interpolation. Additional details and references on the mentioned algorithms can be found here [51,52]. The obtained displacements, given the reference system integral with the cell centroid, can be interpreted as local cell deformations.

Given the obtained vector fields evolving in time, 3-time varying sequences $x_{\mu }\left(t_{i}\right)$, $y_{\mu }\left(t_{i}\right)$, and $\theta_{\mu }\left(t_{i}\right)$ for each ROI are obtained by spatial averaging the individual vector components, and corresponding phase, at each frame, as

$$x_{\mu }\left(t_{i}\right)=\frac{\sum_{n=1}^{N}\sum_{m=1}^{M}u_{t_{i}}\left(n,m\right)}{N\cdot M}$$ (5)

$$y_{\mu }\left(t_{i}\right)=\frac{\sum_{n=1}^{N}\sum_{m=1}^{M}v_{t_{i}}\left(n,m\right)}{N\cdot M}$$ (6)

To derive information on local cell orientations, for each frame, the phase field, $\theta_{t_{i}}\left(n,m\right),$ at time $t_{i}\;$ is computed as

$$\theta_{t_{i}}\left(n,m\right)={\text{tanh}}^{-1}\left[\frac{v_{t_{i}}\left(n,m\right)}{u_{t_{i}}\left(n,m\right)}\right]$$ (7)

Then, the average time-varying phase, $\theta_{\mu }\left(t_{i}\right)\epsilon \left[-1,\;1\right],$ is extracted as

$$\theta_{\mu }\left(t_{i}\right)=\frac{\sum_{n=1}^{N}\sum_{m=1}^{M}\text{cos}\theta_{t_{i}}\left(n,m\right)}{N\cdot M}$$ (8)

This step summarizes the information on the local deformations and orientations and also counteracts the reduction in the accuracy of the PIV-based displacement estimates possibly happening in the presence of microtexture images.

The relative variations, $∆\theta \left(t_{i}\right)=\theta_{\mu }\left(t_{i}\right)-\theta_{\mu }\left(t_{1}\right)$, $∆x\left(t_{i}\right)=x_{\mu }\left(t_{i}\right)-x_{\mu }\left(t_{1}\right)$, and $∆y\left(t_{i}\right)=y_{\mu }\left(t_{i}\right)-y_{\mu }\left(t_{1}\right)$, with *I* = 1,2,…,*T*, with reference to the first frame are considered for feature extraction [38].

#### PIV-based descriptors

The time series $∆x\left(t_{i}\right)$, $∆y\left(t_{i}\right)$, and $∆\theta \left(t_{i}\right)$ are seen as samples of distributions of the deformations over time, representative of the cell’s local responses to the electric field at decreasing frequency values. Indicators of dispersions and shape, i.e., SD, mean absolute deviation, skewness, kurtosis, and maximum and minimum values, of the distributions have been chosen, as summarized in Table S2, to quantify the frequency-dependent statistical characteristics of the cell local responses.

In addition, the approximate entropy [53,54], ApEn, has been quantified, for each of the 3-time series, as indicator of regularity that is, in our case, related to the tendency of the cell to exhibit local displacement (either deformations or rotations) relative to the principal motion, i.e., the centroid motion.

Given a generic time series u($t_{i}$), with *I* = 1,2,…,*N*, of samples equally spaced over time, combinations of 2 sets of delayed blocks, *p*(i) = {*u*($t_{i}$), *u*($t_{i+1}$),…, *u*($t_{i+m-1}$) and *q*(j) = {*u*($t_{j}$), *u*($t_{j+1}$),…, *u*($t_{j+m-1}$) of length *m* (*m* = 2 in our case), are extracted from *u*, with *I* = 1:*N*, and compared to each other as

$$C_{i}^{m}\left(r\right)=\sum_{j=1,j\neq i}^{N-m+1}↿\left({\|}_{p\left(i\;\right)-q\left(j\right)}\|\infty <r\right)$$ (9)

where the symbol ↿ corresponds to the characteristic function, which counts one if the given condition (set as the argument of the function) is verified and 0 otherwise, and ${\left\|x\right\|}_{\infty }=\underset{k}{\text{max}}|x_{k}|.$ Therefore, $C_{i}^{m}\left(r\right)$ corresponds to the number of blocks $q\left(j\right)$, with *j* ≤ *N* − *m* + 1, which are similar to the $p\left(i\right)$ block at the resolution *r*, with *r* radius of similarity.

Then, the ApEn of u is defined as

$$\text{ApEn}\left(r\right)=\phi^{m}\left(r\right)-\phi^{m+1}\left(r\right)$$ (10)

with

$$\phi^{m}\left(r\right)=\frac{1}{N-m+1}\text{log}\left[C_{i}^{m}\left(r\right)\right]$$ (11)

In this work, r=0.2·σ, with σ the SD of the values of the time series *u*.

The statistical descriptors and the approximate entropy are extracted from $∆\;x\left(t_{i}\right)$, $∆y\left(t_{i}\right)$, and $∆\;\theta \left(t_{i}\right)$ associated to each cell, for a total of 21 descriptors. The complete set of descriptors extracted is summarized in Table S2.

### Feature selection and classification

As shown in Fig. 4B and C, a preliminary filter-type feature reduction is performed, at the training level, by selecting those features having a multiclass area under the receiver characteristic curve (${\mathrm{AUC}}_{m}$) defined as

$$\begin{array}{c} {\mathrm{AUC}}_{m}=\underset{i,j}{\text{max}}\left\{\text{max}\left[{\mathrm{AUC}}_{\left\{C_{i}\;\mathrm{vs}\;C_{j}\right\}},1-{\mathrm{AUC}}_{\left\{C_{i}\;\mathrm{vs}\;C_{j}\right\}}\right]\right.;\text{max}\\ \left.\left[{\mathrm{AUC}}_{\left\{C_{i}\;\mathrm{vs}\;\mathrm{all}\right\}},1-{\mathrm{AUC}}_{\left\{C_{i}\;\mathrm{vs}\;\mathrm{all}\right\}}\right]\right\} \end{array}$$ (12)

higher than a given threshold, th, where $C_{i}$ and $C_{j}\;$ indicate the ith and jth class, respectively, with i≠j, so that all possible binary comparative scenarios are taken into account. The values obtained for the AUC in the individual comparative scenarios were also considered to assess the discriminant capability of individual features. Regarding the filtering procedure, given the different number of features present in the 2 subsets of descriptors, i.e., relative to the centroid motion and to the local displacements, 2 threshold values, th=0.6 and th=0.75, are used, respectively, to balance the 2 informative contributions. The obtained features are automatically selected with an SFS algorithm [44]. An automatic stopping criterion with a tolerance equal to 10^−6^ has been included to consider reductions of the classification error relative to the average of the 5 previous steps of iteration. For SFS, within each training subset of patients, a custom cross-validation strategy in leave-one-patient-out (LOPO) was implemented to assess the discriminant capabilities of the features with reference to unknown patients. This ensured that the selected features are capable of generalizing to unknown patients, representing their biological profile in terms of endometrial’s receptivity/selectivity. The selected features are used for training a linear discriminant analysis (LDA) classifier [55] model in a single-cell 3-class scenario of CTRL, RIF, and uRPL (Fig. 4D).

### Study subjects

The present study was carried out in accordance with the Declaration of Helsinki, modified in Tokyo 2004, and was approved by the Institutional Review Board (IRB) of Policlinico Tor Vergata University Hospital (protocol number: 90/18). All women gave their informed written consent to participate in the study. Recruited patients included women attending the Gynaecology and Obstetrics unit of the Policlinico “Tor Vergata” University Hospital and the Physiopathology of Reproduction and Andrology complex operative unit of the Sandro Pertini Hospital.

A total of 9 patients in mid-secretory phase of the menstrual cycle were enrolled and stratified in 3 groups according to their clinical history:

• Fertile group (*n* = 3) with at least one pregnancy at term without complications and no miscarriages.
• RIF group (*n* = 3) with at least 3 failed in vitro fertilization (IVF) attempts with good quality embryos [35]. The grading criteria proposed by the 2011 European Society of Human Reproduction and Embryology (ESHRE) Istanbul Consensus and Gardner were used for the morphokinetic assessment of embryo quality. Patients underwent hysteroscopy during the routine diagnostic workup used to identify potential endometrial causes of RIF.
• uRPL group (*n* = 3) with at least 2 or more pregnancy loss before 24 weeks of gestation, according to the ESHRE guidelines 2022 [36], and for which no causes of miscarriage could be identified after the routine clinical workup.

All the selected women had regular cycles of normal cycle length (28 ± 4 days) monitored by ultrasound imaging. Endometrial samples in mid-secretory phase were collected at day 8 after the luteinizing hormone (LH) surge determined by a urinary test; at the time of sampling, ovulation was confirmed by concomitant ultrasound imaging. The clinical characteristics of the enrolled women are reported in Table 1.

**Table 1.** Characteristics of patients.

	Fertile (*n* = 3)	RIF (*n* = 3)	uRPL (*n* = 3)	*P* value
Age	39 ± 2.6	38 ± 3.1	38.7 ± 3.2	ns
Number of transfers	0.0	4.5 ± 0.7		0.0012
β− (transfer failed)	0.0	3.5 ± 0.7		0.0026
β+	0.0	1 ± 0.0		ns
Number of miscarriages	0.0		2.33 ± 0.5	0.0022

Values are expressed as mean ± SD

### Isolation and culture of primary endometrial stromal cells

Cell cultures were developed from endometrial biopsies using the protocol described by Lacconi et al. [56]. Briefly, tissues were washed in saline solution, cut into 1-mm^3^ pieces, and incubated in calcium- and magnesium-free Hanks’ balanced salt solution (Merck, Darmstadt, Germany) containing 0.25% collagenase (from *Clostridium histolyticum*, catalog C2674, Merck, Darmstadt, Germany), 0.02% deoxyribonuclease I (type IV: from bovine pancreas, Merck, Darmstadt, Germany), and 0.1% hyaluronidase (Merck, Darmstadt, Germany) for 20 min at 37 °C in a humidified atmosphere of 5% CO_2_ in air. Following digestion, the suspension was left to sediment, and the supernatant was collected and passed through 100-μm and then 20-μm mesh filters (pluriSelect, Leipzig, DE) to eliminate any indigested material. The flow through was collected and centrifuged for 5 min at 1,500*g*. The pellet was washed twice in PBS (Corning, New York, USA) and incubated with Red Blood Cell lysis buffer (Roche, Basel, Switzerland) to remove red blood cells. Cells were finally washed in PBS and cultured in phenol red-free RPMI 1640 (Merck, Darmstadt, Germany) supplemented with 10% charcoal-stripped fetal bovine serum (Merck, Darmstadt, Germany), 2 mM l-glutamine, 50 U/ml penicillin, 50 μg/ml streptomycin (Lonza, Milan, Italy), and 0.1 mM sodium pyruvate (Merck, Darmstadt, Germany) at 37 °C in a humidified atmosphere of 5% CO_2_ in air. Once cells reached confluence, they were used for ODEP, gene, and protein expression analyses.

### Gene and protein expression analysis

RNA was extracted from all cell cultures (*n* = 9) using the RNeasy micro kit spin column (Qiagen, Hilden, Germany, catalog no. 74004) according to the manufacturer’s protocol. Specific intron-spanning primers for *NOTCH1*, *EGFL7*, and *GLUT3* were designed using Primer Express software (Applied Biosystems in Life Technologies, Monza, Italy) or the online tool Primer3. Differences in gene expression were quantified using the 2^−DCt^ method normalized to the *RPL17* housekeeping gene. For all used primer pairs, primer efficiency was tested by performing a standard curve. In addition, Western blot analysis was performed on endometrial stromal cells to assess the protein expression of NOTCH1. Further details are included in Sections S5 and S6, and primer sequences are listed in Table S3.

### Statistical analysis

Data are expressed as mean + SEM or SD. Data were analyzed by using either parametric Student’s *t* test or one-way analysis of variance (ANOVA) test, according to the results obtained after variance analysis using Brown–Forsythe or the *F* test, as appropriate. Bonferroni’s test was used for post hoc multiple comparisons among groups after ANOVA. The software used was GraphPad Prism 8. Differences were considered significant at *P* < 0.05.

### Data analysis and cross-validation

A total of 474 TLM videos (137 CTRL, 158 RIF, and 179 RPL) was analyzed. The number of cells monitored in each video varied from 1 to 6, depending on the cells collected during the trapping step. The total number of analyzed cells was 1,106, with 344 cells from the CTRL subjects, 348 cells from the RIF patients, and 414 cells from the RPL patients.

Validation of the results was performed with LOPO cross-validation: All cells belonging to the same patient were left out in each testing round to simulate a realistic operative scenario. Features were selected using a customized criterion that minimizes the classification error of an LDA classifier [55] over nested LOPO partitions of the training set. Discriminant capability of individual features was assessed in 2 class scenarios in terms of AUC and with 2-sample Kolmogorov–Smirnov test. Results were evaluated at the test level in terms of balanced accuracy of classification and percentage of correct classification of individual categories. We have also tested support vector machine (SVM) classifiers with different kernel options obtaining lower or similar results than those obtained with the LDA. With SVM, the best result was obtained with a linear kernel, confirming that the limited number of samples does not favor the use of more complex models in this case, especially in LOPO scenarios. We report the results relative to LDA, also in light of the explainable nature of the feature contributions to the principal components of the model.

At the patient level, the contribution of the local displacements was considered in the following experimental scenario. With the classification result obtained, in test rounds of LOPO cross-validation with features automatically selected in the training data, we randomly sampled a group of cells belonging to the same patient. Majority voting was performed with the group of selected cells to derive the final assignment. For each selected group at an increasing number of cells, 1,000 Monte Carlo repetitions without replacement were performed on the available data with a random selection of cells from the complete set.

## Results

In this section, the obtained results will be described. The ODEP-based single-cell responses have been characterized by complementing the electrokinetic responses of the cell centroid with the electrokinetic responses of the local deformations and orientations, as a consequence of the applied time-varying signal. An automatic system was designed to extract synthetic descriptors and used to derive final assessment via machine learning at both the single-cell and patient levels. All findings have been accompanied by ad hoc biological analysis performed to support our conclusions.

### Extraction of the electrokinetic responses of the cell centroid

**Fig. 5.**
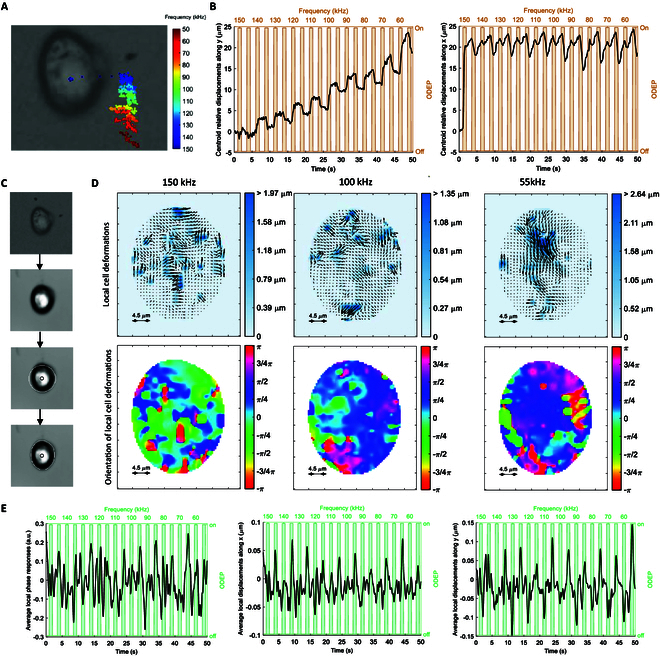
Steps for the derivation of cell motion characteristics in response to ODEP at decreasing frequency values. (A) Cell tracking. (B) Time series of the relative centroid displacements along the *x* and *y* direction over time. ODEP stimuli and corresponding frequency values are indicated in orange. (C) Derivation of the motion area: original ROI centered at the centroid of the cell; cumulative motion response, circular area of radius estimated via CHT; cell motion area, *bw*_cell_ (white border). The boundary area, *bw*_bound_, and the cytoplasmatic area, *bw*_cyt_, are also indicated with 2 different shadow intensities. (D) Local cell deformations (first row) and orientation (second row) for ODEP frequency values of 150, 100, and 55 kHz. (E) Smoothed time series of the average local phase responses and of the average local displacements along the *x* and *y* directions relative to the first frame. ODEP stimuli and corresponding frequency values are indicated in green.

Fig. 5 shows the results of the derivation of the cell electrokinetic responses for a CTRL case example. First, the trajectory of the cell centroid is tracked along time. Given the alternate activation of the 2 electrodes, the cell exhibits an oscillatory motion along the different ODEP frequency values, as indicated by the color code (see Fig. 5A). Both components of the motion in the 2D plane along the *x* and *y* directions (reported in terms of relative contributions in Fig. 5B) are considered and relevant for the subsequent spectral analysis based on WST.

### Extraction of the electrokinetic responses of deformations and orientation

The second contribution is given by the local electrodeformations and orientations superimposed on the principal motion of the cell. Fig. S1 illustrates a set of ROIs at the initial frame of the time-lapse acquisitions containing individual cells from different patients and each of the 3 categories investigated. The results of the steps for the extraction of the cell motion area are shown in Fig. 5C: Starting with the computation of the cumulative image representation, the algorithm proceeds with the initialization given by a circular region of radius estimated via the CHT, and ends with the determination of the final cell motion area, ${\mathrm{bw}}_{\text{cell}},$ and associated boundary area, ${\mathrm{bw}}_{\text{bound}}$, and the cytoplasmatic area, ${\mathrm{bw}}_{\mathrm{cyt}}$, indicated with 2 different shadow intensities in the figure. From the defined area, local cell deformations (first row) and orientation of local cell deformations between consecutive frames (second row) for ODEP frequency values of 150, 100, and 55 kHz are reported (see Fig. 5D). From such vector fields evolving in time, series of the average local phase responses and of the average local displacements along the *x* and *y* directions are derived, as shown in Fig. 5E. All 3-time series show the presence of peaks in correspondence with the ODEP stimuli, with lower or higher amplitude depending on the ODEP frequency, highlighting a relationship with the external spatial distribution of the electric field. The 5-time series represented 2 in Fig. 5B and 3 in Fig. 5E are used as electrokinetic fingerprints for further characterization.

### Comparative analysis and contribution of the local deformations to the accuracy

**Fig. 6.**
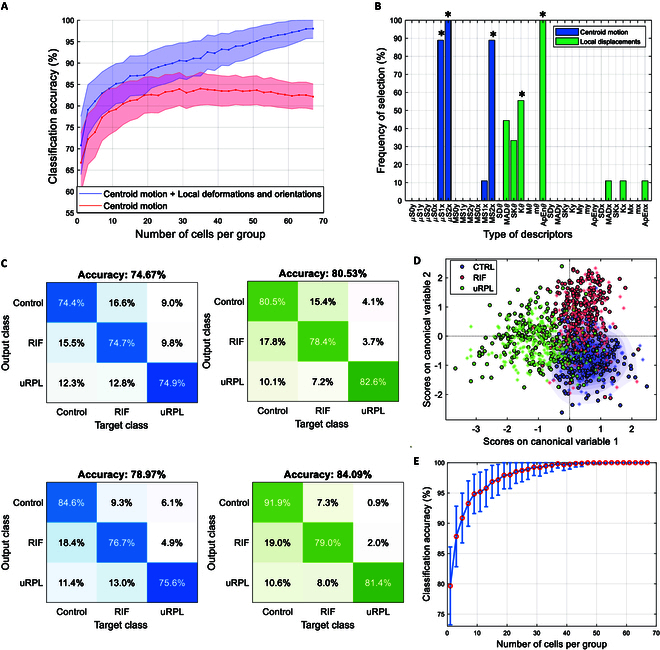
Obtained results. (A) Results obtained in LOPO cross-validation with varying number of cells per subject. Curves and shadows indicate, respectively, average and SD accuracy values obtained with the sole information from the cell centroid motion (in red) and with the inclusion of information from local cell displacements (in blue). (B) Frequency of selection of the different types of descriptors grouped in descriptors of cell centroid motion (blue) and of the cell local displacements (green). Asterisks indicate the types of features mostly selected. (C) Confusion matrices obtained with the most selected features with the sole information from the cell centroid motion (in blue) and with the inclusion of information from local cell displacements (in green). The first row corresponds to single-cell classification, and the second row corresponds to single-experiment classification (majority voting on single TLM videos). (D) Scores on canonical variables obtained with the combined approach. Circles indicate the samples for the training patients, and asterisks indicate the samples for the test patients (3 in this case). (E) Average and SD accuracy values obtained with the most selected features with 1,000 repetitions of random subsampling of cell groups and majority voting at increasing number of cells per group.

In Fig. 6A, the obtained average (and SD values) of balanced accuracy for the increasing number of randomly sampled cells (1,000 Monte Carlo repetitions) and for the 2 scenarios is reported: the system based on the sole centroid motion (red) and with the system including the contribution of the electrodeformations and orientations (blue). To avoid the selection of the same cell more than one time (random sampling without replacement), the analysis ends up at 67 cells. This choice corresponds to the minimum number of available cells for one of the 9 patients, ensuring that the same number of cells could be sampled for all patients.

The combined approach improves at the increasing number of analyzed cells, up to a balanced accuracy of 0.98 (0.05). Moreover, when all available cells from each patient were considered, the combined approach reached the correct classification for all patients. This was not achievable via the approach based on the sole centroid, which reached a balanced accuracy of 0.82 (0.05), and if all cells were considered, one patient was misclassified. In Fig. 6B, the frequency of selection of the different groups of features is reported, with the asterisks indicating the most selected ones. The results obtained with the sole information from the cell centroid motion and with the inclusion of information from local cell deformations and orientations are compared in Fig. 6C when the most selected features were considered. Confusion matrices are shown for the case of single-cell classification (first row) and of single-experiment classification, i.e., a majority voting is performed on multiple cells belonging to the same TLM video. In both scenarios, an increase of 5% in accuracy is observed when the local displacement contribution is included. Balanced accuracy values of 0.75 and 0.79 were obtained by considering descriptors of the sole centroid motion in a single-cell and single-experiment classification, respectively, with LOPO cross-validation. When descriptors of the local displacements are considered, in addition, accuracy values of 0.81 and 0.84 are obtained, respectively, for the same scenarios.

The scores on canonical variables of the training dataset are illustrated in Fig. 6D and include test results relative to 3 patients (one for each category). Finally, in Fig. 6E, the balanced accuracy obtained with majority voting at the increasing number of cells is shown for the scenario of the most selected features. In this case, with groups of 33 cells, it was possible to reach average accuracy levels of 99.5% (2.4%).

### Explainability of the selected descriptors and classification profiles of single patients

**Fig. 7.**
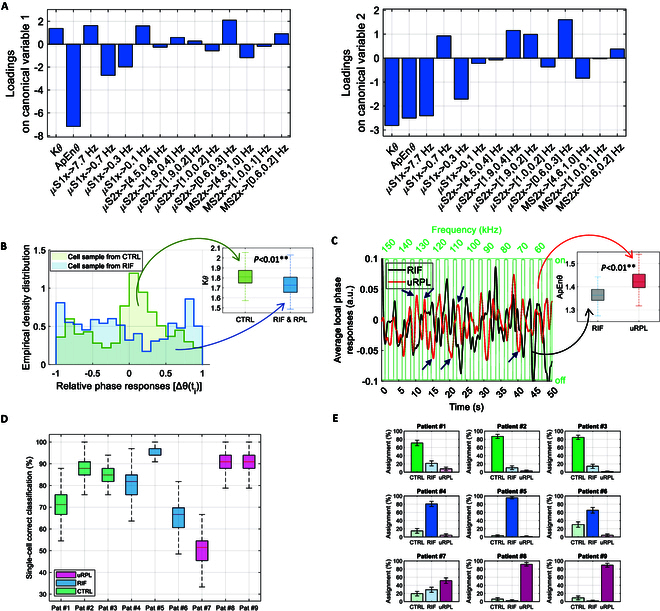
Explainability of the electrokinetic descriptors and classification profiles of single patients. (A) Loadings on canonical variables 1 and 2 obtained with the set of 14 descriptors most selected during the round of LOPO cross-validation. (B) Case example showing the comparison of CTRL versus RIF and uRPL in terms of Kθ and corresponding interpretation. (C) Case example showing the comparison of RIF versus uRPL in terms of ApEnθ and corresponding interpretation. (D) Classification profiles of individual patients in terms of single-cell percentage of correct classification. (E) Classification profiles of individual patients in terms of percentage of assignment of single cells.

The most selected groups of features indicated in Fig. 7A correspond to a total of 14 descriptors, 2 from the local displacements (kurtosis, Kθ, and approximate entropy, ApEnθ, of the relative phase) and 12 from the WST of the centroid motion (all from the *x* components). The contributions of those features to the canonical variables are analyzed in detail by means of the loadings. It can be noted that both the local descriptors, Kθ and ApEnθ, provide the major contributions to the canonical variables. The approximate entropy of the local orientations, ApEnθ, in particular, is the most instrumental to the first canonical variable, which is the one differentiating uRPL from the rest of the classes, and it is also pivotal in canonical variable in discriminating uRPL from RIF. This is confirmed by the corresponding AUC values for this feature: AUC (uRPL versus all) = 0.81, AUC (uRPL versus RIF) = 0.86. Kθ provides, together with ApEnθ and features from the μS1x group, a major contribution to the second canonical variable in the discrimination of RIF. In fact, AUC = 0.72 (uRPL versus RIF) for Kθ, while for the WST-based descriptors of the cell centroid, the 3 most relevant have AUC (uRPL versus RIF) = 0.61 for μS1x7.7 Hz, AUC (uRPL versus RIF) = 0.82 for μS1x0.27 Hz, and AUC (uRPL versus RIF) = 0.84 for μS2x [0.6, 0.26] Hz.

To go into detail about the meaning of the mentioned features, we start with the 2 most relevant ones, the kurtosis and the approximate entropy of the relative phase of the local deformations. In Fig. 7B, the empirical density distribution of the relative phase of the local displacement is represented for 2 sample cells, one from the CTRL group and one from the pathological group (RIF and uRPL). As the parameter is expressed in terms of the cosine of the phase, averaged over the motion area of the cell, values close to the extreme 1 and −1 indicate orientations, on average, toward one of the 2 electrodes. This means that, locally, the displacements are in those directions. Values close to 0 indicate either the average direction of the phase orthogonal to the line connecting the 2 electrodes or contributions that, on average, cancel each other out, like in the absence of stimuli or deformations. It can be noted that the CTRL sample tends to have values close to 0 and to the extreme 1 and −1 over time, while for the pathological samples, the values are distributed more homogeneously. This might indicate a tendency to heterogeneity of the pathological group, reflected by the presence of various directions of deformation, on average, over time, contrary to the CTRL sample whose orientations of deformation tend to be more net. Quantitatively speaking, the difference just described can be expressed in terms of kurtosis, Kθ, that measures the “tailedness” of those distributions [57]. As shown in the corresponding boxplot for the complete set of data, all cells exhibit Kθ lower than 3, i.e., distributions with thicker tails (more extreme values) than the standard gaussian distribution. The analyzed pathological samples (RIF and uRPL), however, tend to have more extreme values and lower kurtosis, indicating that the cells tend to deform less uniformly following the ODEP stimuli. The Kθ distributions of the 2 groups are different with high statistical significance (*P* < 0.01, 2-sample Kolmogorov–Smirnov test).

Regarding the approximate entropy, the characteristics of the time series of the phase responses are illustrated in Fig. 7C for 2 examples, one from the RIF category and one for the uRPL category. It can be observed that the uRPL sample (in red), differently from the RIF sample, exhibits a set of response peaks (indicated with the arrows) outside the windows of ODEP stimulation, conferring major irregularity and unpredictability of the corresponding time series. The RIF case better follows the ODEP stimuli, and this corresponds to increased predictability and regularity. ApEnθ quantifies these aspects and, as shown in the boxplots of Fig. 7D relative to the complete sets of data, shows a significant difference between the 2 categories (*P* < 0.01, 2-sample Kolmogorov–Smirnov test).

The approach developed in the present study is based on the analysis of samples at the single-cell level and allowed the development of a specific classification profile to stratify the 9 patients included in the study. Fig. 7D illustrates the percentage of correctly classified single cells dependently on patient stratification. As reported, the majority of the cells for each group were correctly classified; it is, however, relevant to note that for patient #7 (uRPL), in particular, the percentage of correct classification was lower, indicating a higher heterogeneity of the cells in the sample. This result is more clearly reported in Fig. 7E, where it can be better appreciated that cells from patient #7 have percentage of assignment to CTRL and uRPL.

### Gene and protein expression analysis

**Fig. 8.**
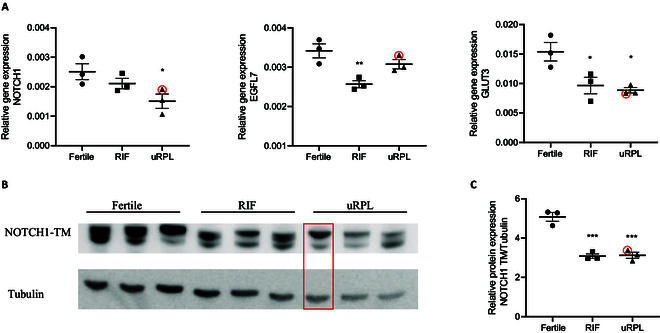
Gene and protein expression analysis in isolated endometrial stromal cells. Patient #7 is highlighted in red. (A) Real-time PCR analysis indicating the reduced expression of NOTCH1, EGFL7, and GLUT3 in RIF and uRPL patients compared to samples from fertile women (*n* = 3). (B) Western blot analysis showing the decreased expression of NOTCH1 in RIF and uRPL patients compared to fertile women. (C) Quantification of Western blot analysis (*n* = 3). *P ≤ 0.05, **P ≤ 0.01, ***P ≤ 0.001 versus fertile women, ANOVA, and Bonferroni test.

To further address whether the ODEP analysis could efficiently contribute to the discrimination of the 3 groups of patients, we performed gene expression analysis for genes whose role in the preparation of the endometrium to embryo implantation has been widely reported and their altered expression has been associated to RIF and/or RPL conditions. The following markers were assessed: NOTCH1, GLUT3, and EGFL7. The expression of the transmembrane receptor NOTCH1 was down-regulated in both RIF and uRPL patients compared to the fertile controls, although statistical significance was only met in the uRPL samples (Fig. 8A). Results were further confirmed by Western blot analysis. As reported in Fig. 8B and C, protein levels of NOTCH1 were significantly lower in both RIF and uRPL samples than in the fertile control samples, confirming that differential expression of NOTCH1 allows to discriminate between fertile patients and patients experiencing reproductive failure. We also assessed the expression of EGFL7, a modulator of the NOTCH signaling pathway that we previously demonstrated to be down-regulated in endometrial stromal cells from RIF and uRPL patients [56]. In line with our previous data, the expression of EGFL7 was down-regulated in both pathological sample groups, although statistical significance was only reached in the RIF sample (Fig. 8A). Similarly, in line with literature data, we also observed a statistically significant decreased expression of glucose transporter GLUT3 in both RIF and uRPL samples (Fig. 8A). In the figure, gene and protein expression results relative to patient #7 are highlighted in red, stressing the profile of the subject.

## Discussion

In this work, using patient-derived primary endometrial stromal cells, we demonstrate for the first time that an ODEP-based robotic system can provide meaningful information into single-cell phenotypes, particularly concerning implantation defects, whose underlying causes remain largely unexplained. The proposed automated platform enables in-flow single-cell micromanipulation and measurement using highly reconfigurable projected electrodes. In fact, by easily varying the ODEP stimuli at decreasing frequency values, the electrokinetic responses of cells have been collected to better elucidate the dynamic patterns in nonstationary conditions.

During the time-lapse acquisitions, each cell undergoes through a series of composite oscillatory motions that can be regarded as the combination of the centroid displacements accompanied by local displacements, resulting in local deformations and reorientations. Such local displacements, being relative to the centroid motion, are lower than those of the centroid itself and localized in cell portions, which respond differently from the electrokinetic point of view. This behavior reflects the fact that cells are heterogeneous, viscoelastic, and not perfectly spherical bodies with local distinct dielectric, morphological, and structural properties.

We demonstrate that the time-varying cell electrokinetic signatures, derived from global and local ODEP-induced displacements, both contribute to the definition of pathological profiles, learned and derived via machine learning techniques. We compared the discriminant capabilities of the system based on the sole information related to the cell centroid, with the combined approach also integrating the local electrodeformations and orientations. In particular, as reported in Fig. 6, we show that, with respect to the system based on the sole cell centroid motion, the inclusion of data from electrodeformation and orientations brought improvements in the classification performance that are almost equally distributed across the 3 classes, with an increase in sensitivity of more than 6% for the uRPL category at the single-cell level. With the canonical variables represented in Fig. 6D, we also showed that some of the cells of the diseased patients are within the area of the CTRL potential (indicated with the blue shadow), suggesting that the abnormal dielectric phenotype is not present in all cells but nevertheless represents the majority of them. To derive a more effective representation of the patients’ outcome, the analysis was extended, with majority voting, on groups of cells from the same subject, which helped increase the accuracy of the system. From the analysis of the patients’ profile in Fig. 7, we can speculate that not all cells exhibit abnormal characteristics and that the final endometrial outcome is represented by the majority of the cell phenotypes rather than the totality, supporting the hypothesis of heterogeneity of the stromal cell population. This also suggests the possibility that the pathological condition arises from an imbalance in the equilibrium among subpopulations. When multiple cells were considered at the patient level, the performance increased by more than 16%, from an accuracy of 0.82 (0.05) up to an accuracy of 0.98 (0.05). Moreover, when all available cells from each patient were considered, the combined approach reached the correct classification of all patients and, in particular, of patient #7 (see Figs. 6A and 7D and E), who would have been otherwise misclassified via our previous approach based on the sole cell centroid [21].

Given the explainable nature of the descriptors, in the “Explainability of the selected descriptors and classification profiles of single patients” section, it was possible to interpret the meaning and the different contributions of the synthetic descriptors to the final assessment. Regarding the automatically selected descriptors, in the combined approach proposed in this study, only the WST descriptors related to the centroid displacements along *x*, i.e., the direction along the flow and along the line connecting the 2 electrodes, have been selected. These results differ from the results obtained in our previous work [21], in which the centroid displacements along *y* were correlated to the cell mass imbalances and dielectric nonuniformities. However, both contributions along *x* and *y* were selected when the information of the centroid motion was considered alone, thus confirming our previous findings. The contribution along the *y* displacement, in the combined approach, has been compensated by the orientation of the local deformations, which seem to provide a more robust representation of cell heterogeneity. The central frequency values associated with the most relevant WST descriptors (indicated by their symbols) can be interpreted in light of the main frequency of oscillation of the cell that is expected to be 0.2 Hz so that the frequency variations relevant to the discrimination of the 3 categories analyzed can be elucidated. These results suggest that the periodicity of the multi-frequency trajectories induced by ODEP stimuli is altered in the pathological conditions.

Regarding the electrokinetics of deformations and orientations, the 2 major contributions to the classification model were given by the kinematic of variations of the local orientations and, in particular, by kurtosis, Kθ, and approximate entropy, ApEnθ (see the “Explainability of the selected descriptors and classification profiles of single patients” section). Descriptors of the orientations provided more robust indications on the cell status than the corresponding amplitude values, which might have been more influenced by confounding factors, e.g., cell radius. ApEnθ, in particular, has been chosen as a descriptor of the dynamic characteristics of the system instead of other metrics in light of its robustness to noise [53] paired with its ability to quantify the amount of regularity and predictability of time series. In this study, ApEnθ resulted to be the most instrumental to the differentiation of uRPL from the rest of the classes, as well as in discriminating uRPL from RIF. This was confirmed by the corresponding AUC values for this feature: AUC (uRPL versus all) = 0.81, AUC (uRPL versus RIF) = 0.86. These results might suggest that the capacity of local portions of the cells to orient toward the alternatively active electrodes varies in the presence of the different pathological profiles. In fact, while the ODEP stimuli are characterized by regular activation patterns (periodical signals), alterations in the regularity and predictability of the orientations of the deformations reflect heterogeneities in the dielectric responses of the local structures of the cells, which are eventually related to their local viscoelastic properties.

In the computation of ApEn, setting *r* = 0.2*σ* acts “the facto” as noise filtering for the input signals and it is considered to be in the range of statistical validity [58]. Also, the length of the signals, which is not lower than 1,000 frames in our study, is consistent with the recommended values in the literature [54]. We performed preliminary tests varying the *r* value in the range [0.1 to 0.4]σ and obtained variations in the accuracy of classification of 1% to 2%. We decided to report the results obtained with the default parameters (without optimization). Additional tests were performed by introducing smoothing spline on the dynamic signals at decreasing values of the smoothing parameter *p* over the range [0.01 to 1]. Lower values of *p* brought improvements in other features (e.g., kurtosis) in terms of individual AUC but decreased the AUC for ApEn, confirming that fast variations in cell shape are pivotal for the discrimination of the 2 categories based on ApEn. With the use of the mostly selected features at different level of smoothing, the accuracy of classification at the single-cell level varied in the range [75% to 82%]. Finally, sensitivity to artifacts was tested by comparing the results obtained in the presence or absence of impulsive noise due to repetitions of the same frame, happening randomly over the course of the time-lapse acquisitions due to the high frame rate. The results obtained at the classification level in the 2 scenarios were comparable, and no significant differences were found. These analyses substantially demonstrated a certain degree of stability of the obtained results with reference to the experimental noise and minor variations in input data.

Overall, local displacements relative to the cell centroid, which result in local cell deformation and reorientation, provide complementary information to the analysis of cell electrokinetics. While electrokinetics primarily focuses on absolute cell motion, the study of these local changes offered additional insights. The findings on cell deformation support the notion that cell plasticity plays a significant role in biological processes, including cell growth and differentiation. The ability of cells to deform when subject to local nonuniformities of the electric field is related to the cell mechanical properties. Endometrial cells, in particular, during the menstrual cycle, respond to mechanical forces to prepare the endometrium for implantation [59]. Alterations in cell deformability have been associated to cell-related diseases (e.g., malaria, sickle cell disease, and cancer) and, in particular, to endometriosis in relation to the Raf-1/Rho/ROCKII pathway, which are key regulators of the cytoskeleton structure [60]. Cell characteristics that determine the receptivity/selectivity equilibrium are still under debate [61]. In this work, we analyzed the expression of 3 transmembrane/surface markers that were differentially expressed in the controls compared to the RIF/RPL samples. These markers were selected based on their relevance to endometrial cell function and their potential impact on cellular behavior in the context of reproductive health. The obtained biological profiles (see the “Gene and protein expression analysis” section and Fig. 8) are in line with previous studies reported in the literature [56]. Particular attention must be placed on patient #7 (uRPL). The assignment from the majority voting on groups of cells from this sample is never correct if the sole information from the cell centroid motion is considered. The inclusion of information from local cell deformation, including a good number of cells, determines an increase in the percentage of correct classification as uRPL, although the percentage of cell assignment to CTRL and uRPL still remains, as shown in Fig. 7E. This behavior is further supported by the experimental results reported in the “Gene and protein expression analysis” section where the values of relative gene and protein expressions are closer to RIF or CTRL groups (see Fig. 8). Although the biological profiles of the analyzed patients can be heterogeneous, our results suggest that the differential expression of the selected surface markers in RIF/RPL samples compared to controls may lead to alterations in the membrane structure, charge, and mechanical properties of the cells, which in turn affect their electrokinetic behavior. For example, changes in membrane elasticity and dielectric properties can influence the cell interaction with electric fields, leading to distinct electrokinetic profiles.

The results presented in this work represent, to our knowledge, the first proof of ODEP-based characterization of primary cells from endometrial biopsies for diagnostic purposes. This is a preliminary yet fundamental step to the validation of the systems in real clinical scenarios, which account for the complexity and heterogeneity of cells in humans. It is important to stress that the patient cohort used is limited and further tests and validation steps need to be performed to assess the capability of the platform in real clinical practice. At this regard, we must refer to current international guidelines, like EN ISO 13485, and related requirements [62,63]. To reach appropriate scalability on larger patient cohorts, we expect the machine learning models to be upgraded with nonlinear or deep architectures, capable of handling bigger and diversified data. These aspects will be certainly addressed in our future studies. We believe that ODEP technology could be easily integrated into clinical settings, in light of its flexibility and programmability [7], to complement histopathological examination. Additionally, the design of high-throughput processing and automation could streamline its adoption in routine clinical diagnostics. One key advantage of ODEP is the small number of cells required for analysis, enabling its use on residual material from routine diagnostic procedures. We also recognize that successful clinical implementation requires not only diagnostic accuracy but also the ability to monitor patients over time, including during repeated pregnancies and different stages of the reproductive process. Future research could incorporate longitudinal studies, integration with other diagnostic tools, and the potential for updates to ODEP-based protocols.

In conclusion, the present study represents proof of principle of the use of ODEP analysis for the classification of patient-derived endometrial stromal cells, which could be exploited to help clinicians to stratify patients experiencing reproductive failure. One main limitation of our results is represented by the low number of patients analyzed; however, it is still enough to reach statistical significance. Further studies are warranted to test the robustness and generalizability of the approach. Nevertheless, positive indicators of the effectiveness of the proposed methods stem from the fact that the patients were sourced from 2 hospitals and that all results were validated using LOPO scenarios. This lays the foundation for future applications of ODEP-based robotic systems to support cell characterization in the presence of multifactorial defects.

## Data Availability

All the data are available upon request on the corresponding author.
