# Supplementary material for: ODEP-Based Robotic System for Micromanipulation and In-Flow Analysis of Primary Cells
Source: Cyborg Bionic Syst. 2025 Mar 6;6:0234. doi: 10.34133/cbsystems.0234 (PMC12087799; doi:10.34133/cbsystems.0234)
Supplement: Supplementary 1 — Supplementary Text Fig. S1 Tables S1 to S3 Movies S1 to S3 [file cbsystems.0234.f1.zip › ODEP_2.1_supplementary_v5.docx]

**Supplementary Materials**

ODEP-based Robotic System for Micro-Manipulation and In-Flow Analysis of Primary Cells

ODEP-based Robotics for Cell Phenotyping

Joanna Filippi^1,2,#^, Paola Casti^1,2,#^, Valentina Lacconi^3^, Gianni Antonelli^1,2^, Michele D’Orazio^1,2^, Giorgia Curci^1,2^, Carlo Ticconi^6^, Rocco Rago^7^, Massimiliano De Luca^4^, Alessandro Pecora^4^, Arianna Mencattini^1,2^, Steven L. Neale^5^, Luisa Campagnolo^3^, Eugenio Martinelli^1,2^

^1^ Department of Electronic Engineering, University of Rome Tor Vergata, Via del Politecnico 1, 00133 Rome, Italy

^2^ Interdisciplinary Center for Advanced Studies on Lab-on-Chip and Organ-on-Chip Applications (ICLOC), Via del Politecnico 1, 00133 Rome, Italy

^3^ Department of Biomedicine and Prevention, Tor Vergata University, Rome, Italy, 00133 Rome, Italy

^4^ Italian Nation Research Council (CNR), Rome, Italy

^5^ James Watt School of Engineering, University of Glasgow, Glasgow, UK

^6^ Department of Surgical Sciences, Section of Gynecology and Obstetrics, University of Rome Tor Vergata, Rome, Italy

^7^ Department of Gender, Parenting, Child and Adolescent Medicine, Physiopathology of Reproduction and Andrology Unit, Sandro Pertini Hospital, Rome, Italy

# equally contribution

* corresponding author (martinelli@ing.uniroma2.it)

**S1. Fabrication of the ODEP-based device**

The chamber of the device is constituted by two layers of ITO-coated one of them coated by a-Si by leaving free 25 mm to create the electric contact (L: 50 mm, W: 25 mm, H: 600 nm). The thickness of ITO is 200 nm. The above-mentioned layers were bonded together with a double-sided adhesive tape channel (467 MP transfer tape 3M™ Maplewood, US) by pressing with a hydraulic press (Betafer, Beta 3027) at 5 bar and 70 °C for 15 min. Two holes were realized by drilling the top layer (Dremel™, USA) to create its inlet and outlet. The chip was fastened on the microscope stage insert via two polymethyl-methacrylate (PMMA) layers and fabricated by laser cutting 3-mm-thick (TroGlass Clear, Trotec Laser Inc., Austria) and 0.8-mm-thick (TroLase, Trotec Laser Inc., Austria) PMMA sheets. The latter also acts as support for a copper-based adhesive (AT526, Advance Tapes International, Leicester, UK) that electrically contact the top ITO-coated slide of the device. A 3 mm-thick PMMA piece was fabricated (L: 25mm, W: 3 mm) and covered with copper tape to electrically contact the bottom ITO-coated glass slide. The fluidic connections were realized by means of two PMMA layers. The first one, covered on both sides with adhesive tape, has a thickness of 0.8 mm (TroLase, Trotec Laser Inc., Austria) and acts mainly as insert for the top ITO-coated glass slide and the second one, with a thickness of 3 mm (TroGlass Clear, Trotec Laser Inc., Austria), hosts the luers (Fluidic 331 Male Mini Luer, Microfluidic ChipShop). The two PMMA layers were first bonded together by pressing at 50 bar and after bonded to the device by pressing at 5 bar and 70 °C for 15 min.

**S2. Extraction of cell centroid trajectories**

At every frame, *Cell Hunter* [39], [40], [41], [42] detects the circular-shaped objects using the circular Hough transform (CHT) [43] and, by means of the Munkres algorithm [44], it links the centroid of the circular regions, associated with the cells, at each time-point by solving an optimal subpattern assignment problem. Further details can be found in the work by Comes et al. [42]. Each trajectory of coordinates ($x\left( t_{i} \right),y\left( t_{i} \right)$), with i = 1, 2, …, T, with T equal to the total number of frames, is normalized with respect to the position at the starting point at time $t_{1}$ and decomposed in its x and y directions that we called relative displacement $X_{s}$ along the x-direction (corresponding to flow direction) and defined as follows:

$X_{s}(t_{i})=\Delta x\left( t_{i} \right)=x\left( t_{i} \right)-x\left( t_{1} \right)$ (1)

and relative absolute displacement $Y_{s}$ along the y-direction (corresponding to the direction orthogonal to the flow) and defined as follows:

$Y_{s}(t_{i})=|\Delta y\left( t_{i} \right)|=|y\left( t_{i} \right)-y\left( t_{1} \right)|$. (2)

Positive values of the $X_{s}$ displacement are intended toward the virtual pattern 1 (right) whilst negative values are intended toward the virtual pattern 2 (left). The absolute value makes the displacements invariant to the direction of the motion which depend on the initial cell positioning relative to the centre of the electrode. To reduce noise, the obtained displacements are pre-processed via smoothing spline.

**S3. WST-based descriptors**

This information can be extrapolated by means of the WST [26], [27] through a cascade of signal decomposition to characterize the non-stationarity of the cell motion [18]. At zero step, we obtain the zero-scattering coefficient by decomposing both $X_{s}$ and $Y_{s}$, that we name $f(t_{i})$ for conciseness, through a deconvolution as follows:

$S_{0}f\left( t_{i} \right)=f(t_{i})*\phi_{J}(t_{i})$, (3)

where $\phi_{J}\left( t_{i} \right)$ is a low-pass filter at invariance scale S_T_ = 0.5∙T, with T equal to the signal duration. This process brings to a loss of information at high frequency that can be restored in two subsequent steps. First the input signal$f(t)$ was convolved with a set of dilated versions of the mother Morlet wavelet ψ(t). Multiscale filter banks, $\left\{ \psi_{j_{k}} \right\}_{j_{k}\in\Lambda_{k}}$, with $k\mathbb{\in Z}$ denoting the $k_{th}$ octave frequency resolution $Q_{k}$, i.e., the number of wavelets per octave, and $\Lambda_{k}$ being the indices of the wavelet family at that frequency resolution, are obtained by dilating the mother wavelet, $\psi\left( t \right)$ and so covering the whole frequencies contained in the signal. Named $\psi_{j_{1}}$, with $j_{1}\in\Lambda_{1}$, the filters bank at the frequency resolution $Q_{1}$. After, by averaging the wavelet modulus transform, we obtain the first-order scattering coefficients as follows:

$S_{1}f\left( t \right)= \left\{ \left| f(t)*\psi_{j_{1}}(t) \right|*\phi_{J}(t) \right\}_{j_{1}\in\Lambda_{1}}$ (4)

For clarity, frequency resolution does not represent the frequency of the ODEP stimuli but the frequency content of the time-varying signals.

In cascade, the second-order scattering coefficients can be obtained with the wavelet $\psi_{j_{2}}$, with $j_{2}\in\Lambda_{2},$ at the frequency resolution $Q_{2}$ as follows:

$S_{2}f\left( t \right)= \left\{ \left| \left| f(t)*\psi_{j_{1}}(t) \right|*\psi_{j_{2}}(t) \right|*\phi_{J}(t) \right\}_{j_{2}\in\Lambda_{2}}$. (5)

In the same manner, the higher order scattering coefficients will be:

$S_{m}f\left( t \right)= \left\{ \left| \left| \left| f*\psi_{j_{1}} \right|*\cdots\right|*\psi_{j_{m}} \right|*\phi_{J}(t) \right\}_{j_{m}\in\Lambda_{m}}$ (6)

with $m=3,4\ldots$

In this work we considered only up to the second-order scattering coefficients because the derived wavelet representation will contain the 99% of the signal information. The filter banks used for the first and second order WST have quality factors equal to $Q_{1}=4$ and $Q_{2}=2$ corresponding to 4 and 2 wavelets per octave, respectively, with central frequency values in the range [0.06-9.1] Hz and [0.07-8.3] Hz. The invariance scale S_T_ was set equal to 25 s (500 frames). So, the matrix of the scattering coefficient $\boldsymbol{S}f\in\mathbb{R}^{N_{S}x N_{batch}}$ obtained from each signal$f(t)$ is the following:

$\boldsymbol{S}f=\left[ \begin{matrix} {S_{0}f\left( t \right)}_{N_{S_{0}}x N_{scat}} \\ {S_{1}f\left( t \right)}_{N_{S_{2}}x N_{scat}} \\ {S_{2}f\left( t \right)}_{N_{S_{2}}x N_{scat}} \end{matrix} \right]=\left[ \begin{matrix} {S_{0}f\left( t \right)}_{1x 8} \\ {S_{1}f\left( t \right)}_{23 x 8} \\ {S_{2}f\left( t \right)}_{118 x 8} \end{matrix} \right]$, (7)

where $N_{scat}$ represents the number of scattering coefficients over time for each path and was equal to 8. The total number of scattering paths is 142 ($N_{S}=N_{S_{0}}+N_{S_{1}}+N_{S_{2}}$). By considering the mean and the maximum response over time, i.e., over the $N_{scat}$coefficients of each path, a set of final descriptors is derived as follows:

$\boldsymbol{\mu Sy(c)=}\frac{1}{N_{scat}}\sum_{n=1}^{N_{scat}} \boldsymbol{S}Y_{s}(c,n)$, (8)

$\boldsymbol{\mu Sx(c)=}\frac{1}{N_{scat}}\sum_{n=1}^{N_{scat}} \boldsymbol{S}X_{s}(c,n)$, (9)

$\boldsymbol{MSy(c)=}\max_{n\in N_{scat}} \boldsymbol{S}Y_{s}(c,n)$, (10)

$\boldsymbol{MSx(c)=}\max_{n\in N_{scat}} \boldsymbol{S}X_{s}(c,n),$ (11)

corresponding to a total number of 142x4=568 WST-based descriptors extracted from the relative displacement signals of each cell, as summarized in Table S1.

Table S1. List of wavelet-based descriptors of the cell centroid displacements

| **Symbol** | **Number of descriptors** | **Description** |
| --- | --- | --- |
| $\boldsymbol{\mu}\boldsymbol{S}_{\boldsymbol{0}}\boldsymbol{y}$ | 1 | Average over time of the 0th-order coefficient of centroid displacements along the y-axis |
| $\boldsymbol{\mu}\boldsymbol{S}_{\boldsymbol{1}}\boldsymbol{y}$ | 23 | Average over time of the 1st-order coefficients of centroid displacements along the y-axis |
| $\boldsymbol{\mu}\boldsymbol{S}_{\boldsymbol{2}}\boldsymbol{y}$ | 118 | Average over time of the 2nd-order coefficients of centroid displacements along the y-axis |
| $\boldsymbol{\mu}\boldsymbol{S}_{\boldsymbol{0}}\boldsymbol{x}$ | 1 | Average over time of the 0th-order coefficient of cell centroid displacements along the y-axis |
| $\boldsymbol{\mu}\boldsymbol{S}_{\boldsymbol{1}}\boldsymbol{x}$ | 23 | Average over time of the 1st-order coefficients of centroid displacements along the y-axis |
| $\boldsymbol{\mu}\boldsymbol{S}_{\boldsymbol{2}}\boldsymbol{x}$ | 118 | Average over time of the 2nd-order coefficients of centroid displacements along the y-axis |
| $\boldsymbol{M}\boldsymbol{S}_{\boldsymbol{0}}\boldsymbol{y}$ | 1 | Maximum over time of the 0th-order coefficient of cell centroid displacements along the y-axis |
| $\boldsymbol{M}\boldsymbol{S}_{\boldsymbol{1}}\boldsymbol{y}$ | 23 | Maximum over time of the 1st-order coefficients of centroid displacements along the y-axis |
| $\boldsymbol{M}\boldsymbol{S}_{\boldsymbol{2}}\boldsymbol{y}$ | 118 | Maximum over time of the 2nd-order coefficients of centroid displacements along the y-axis |
| $\boldsymbol{M}\boldsymbol{S}_{\boldsymbol{0}}\boldsymbol{x}$ | 1 | Maximum over time of the 0th-order coefficients of centroid displacements along the y-axis |
| $\boldsymbol{M}\boldsymbol{S}_{\boldsymbol{1}}\boldsymbol{x}$ | 23 | Maximum over time of the 1st-order coefficients of centroid displacements along the y-axis |
| $\boldsymbol{M}\boldsymbol{S}_{\boldsymbol{2}}\boldsymbol{x}$ | 118 | Maximum over time of the 2nd-order coefficients of centroid displacements along the y-axis |

**S4. PIV-based descriptors**

Table S2. List of PIV-based descriptors of the cell local displacements

| **Symbol** | **Description** |
| --- | --- |
| $\boldsymbol{SD\theta}$ | Standard deviation of the distribution of relative variations of PIV phase, ∆θ(t), over time |
| $\boldsymbol{MAD\theta}$ | Mean absolute deviation of the distribution of relative variations of PIV phase, ∆θ(t), over time |
| $\boldsymbol{SK\theta}$ | Skewness of the distribution of variations of relative variations of PIV phase, ∆θ(t), over time |
| $\boldsymbol{K\theta}$ | Kurtosis of the distribution of variations of relative variations of PIV phase, ∆θ(t), over time |
| $\boldsymbol{M\theta}$ | Maximum of the distribution of variations of relative variations of PIV phase, ∆θ(t), over time |
| $\boldsymbol{m\theta}$ | Minimum of the distribution of variations of relative variations of PIV phase, ∆θ(t), over time |
| $\boldsymbol{ApEn\theta}$ | Approximate entropy of the sampled relative variations of PIV phase, ∆θ(t), over time |
| $\boldsymbol{SDy}$ | Standard deviation of the distribution of relative variations of PIV displacements along y, ∆y(t), over time |
| $\boldsymbol{MADy}$ | Mean absolute deviation of the distribution of relative variations of PIV displacements along y, ∆y(t), over time |
| $\boldsymbol{SKy}$ | Skewness of the distribution of relative variations of PIV displacements along y, ∆y(t), over time |
| $\boldsymbol{Ky}$ | Kurtosis of the distribution of relative variations of PIV displacements along y, ∆y(t), over time |
| $\boldsymbol{My}$ | Maximum of the distribution of relative variations of PIV displacements along y, ∆y(t), over time |
| $\boldsymbol{my}$ | Minimum of the distribution of the sampled relative variations of PIV displacements along y, ∆y(t), over time |
| $\boldsymbol{ApEny}$ | Approximate entropy of the distribution of relative variations of PIV of PIV displacements along y, ∆y(t), over time |
| $\boldsymbol{SDx}$ | Standard deviation of the distribution of relative variations of PIV displacements along y, ∆y(t), over time |
| $\boldsymbol{MADx}$ | Mean absolute deviation of the distribution of relative variations of PIV displacements along y, ∆y(t), over time |
| $\boldsymbol{SKx}$ | Skewness of the distribution of relative variations of PIV displacements along x, ∆x(t), over time |
| $\boldsymbol{Kx}$ | Kurtosis of the distribution of relative variations of PIV displacements along x, ∆x(t), over time |
| $\boldsymbol{Mx}$ | Maximum of the distribution of relative variations of PIV displacements along x, ∆x(t), over time |
| $\boldsymbol{mx}$ | Minimum of the distribution of relative variations of PIV displacements along x, ∆x(t), over time |
| $\boldsymbol{ApEnx}$ | Approximate entropy of the sampled relative variations of PIV displacements along x, ∆x(t), over time |

**S5. Gene expression analysis**

RNA quality was assessed by evaluating the presence of ribosomal RNA bands in agarose gels and quantified using Nanodrop spectrophotometer ND-1000 (Euroclone). 1µg of RNA was reverse transcribed using random primers and the QuantiTect Reverse Transcription Kit (Qiagen, Hilden, Germany) following the manufacturer’s specifications. Gene expression was assessed using iTaq Universal SYBR Green Supermix (Biorad Laboratories, Hercules, CA, USA). Real-time PCR was performed in the LightCycler 96 Real Time PCR System (Roche Diagnostics GmbH, Mannheim, Germany).

| Gene | Primer sequence |
| --- | --- |
| NOTCH1 (forward) | 5′-GCGGGATCCACTGTGAGAA -3′ |
| NOTCH1 (reverse) | 5′-CCGTTGAAGCAGGAGCTCTCT-3′ |
| EGFL7 (forward) | 5′-TCTGCAGACGGTACACTCTGTGT-3′ |
| EGFL7 (reverse) | 5′-TCTGCACTTCTTCCTTCATTGC-3′ |
| GLUT3 (forward) | 5′-TGCCTTTGGCACTCTCAACCAG -3′ |
| GLUT3 (reverse) | 5′-GCCATAGCTCTTCAGACCCAAG -3′ |
| RPL17 (forward) | 5′- ACGAAAAGCCACGAAGTATCTG -3′ |
| RPL17 (reverse) | 5′- GACCTTGTGTCCAGCCCCAT -3′ |

Table S3. Primer sequences

**S6. Western blot**

Endometrial stromal cells were homogenized in lysis buffer (50 mM Tris–HCl pH 7.5, 150 mM NaCl, 0.5% NP-40, 5 mM ethylenediaminetetraacetic acid (EDTA), 0.5% sodium deoxycholate, 1 mM phenylmethylsulfonyl fluoride, 20 mM b-glycerophosphate, 1 mM sodium orthovanadate) containing EDTA-free protease inhibitor cocktail (Roche, Penzberg, Germany). Bradford assay was used to determine protein content. Forty micrograms of protein extract were separated by electrophoresis on NuPAGE 4-12% Bis-Tris gels (Invitrogen) and transferred to polyvinylidene difluoride (PVDF) Transfer Membrane HybondTM (Amersham Biosciences). Membranes were blocked with 5% (w/v) non-fat dry milk in Tris-buffered saline (TBS) containing 0.1% (v/v) Tween 20 (TBS/T) for 1 h at room temperature and incubated overnight at 4°C with rabbit anti-NOTCH1 antibody (clone D6F11, Cell Signaling, MA, USA, cat BK4380SCST, 1:1000) or mouse anti-GAPDH (clone 6C5, Santa Cruz, CA, USA, cat. sc-32233, 1:2000). Horseradish peroxidase conjugated secondary anti-rabbit and anti-mouse antibodies (Amersham Biosciences) were diluted in 5% (w/v) non-fat dry milk containing TBS/T (1:10000 and 1:5000, respectively) and incubated for 1 h at room temperature. Bands were detected using LiteAblot Turbo chemiluminescent substrate (Euroclone, Pero, Italy) according to the manufacturer’s protocol. Densitometric analysis of the bands was performed using ImageQuant LAS 4000 mini (GE Healthcare, Chicago, USA).

**Supplementary Figure S1**


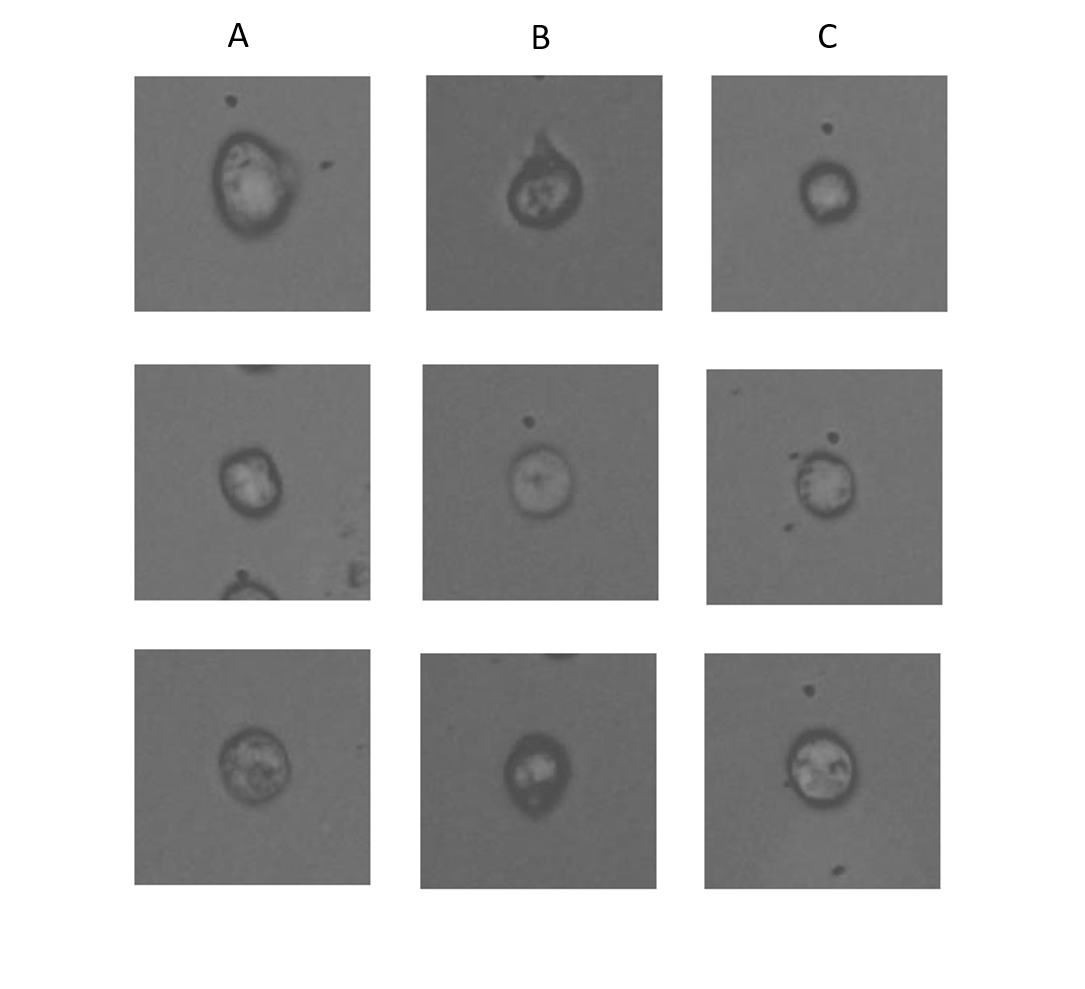


Fig. S1. Examples of regions of interest containing a single cell relative to different patients from each of the three biological category A) CTRL B) RIF C) uRPL.

**Movies S1 to S3**

**Supplementary Video S1**: screen video recording showing examples of the trapping procedure of human endometrial stromal cells with superimposed electrodes at 10x magnification.

**Supplementary Video S2**: video showing an example of human endometrial stromal cell in the reference system integral with the cell centroid at 10x magnification during the ODEP stimuli at varying frequency.

**Supplementary Video S3**: video showing the time-varying vector fields of the electrodeformations obtained for the case example of Video S2.
